# Intra-articular hyaluronic acid and corticosteroids in the treatment of knee osteoarthritis: A meta-analysis

**DOI:** 10.3892/etm.2014.2131

**Published:** 2014-12-15

**Authors:** FANG WANG, XIJING HE

**Affiliations:** Orthopedics Department, Second Affiliated Hospital, College of Medicine, Xi’an Jiaotong University, Xi’an, Shaanxi 710004, P.R. China

**Keywords:** osteoarthritis, knee, intra-articular, hyaluronic acid, corticosteroids, meta-analysis

## Abstract

The aim of the present study was to evaluate the therapeutic effect of intra-articular hyaluronic acid (HA) in comparison to corticosteroids (CS) for knee osteoarthritis (OA). The data sources included PubMed, EMBASE, The Cochrane Central Register of Controlled Trials and hand searched reviews. Randomized controlled trials that reported the effects of intra-articular HA and CS in the treatment of knee OA were selected based on specific inclusion criteria. A meta-analysis was performed for the visual analog scale (VAS), Lequesne index, Knee Society Clinical Rating System (KSS), maximum flexion and adverse events of knee OA. Sensitivity analysis was also conducted to avoid bias. The seven eligible trials included 583 participants and the majority of the trials were of high quality. After one month, the mean difference in the VAS was 1.66 [95% confidence interval (CI); −0.90, 4.23), indicating equal efficacy for HA and CS. However, after three months, the mean difference was −12.58 (95% CI; −17.76, −7.40), while after six months, the difference was −9.01 (95% CI; −12.62, −5.40), favoring HA. For the additional indicators, including the Lequesne index, the KSS, maximum flexion and adverse events, no statistically significant differences were observed between the two treatment approaches for knee OA. Therefore, the results of the meta-analysis highlight a therapeutic trajectory for intra-articular HA in knee OA pain, as compared with CS, over six months post-intervention. After one month, the two approaches exhibited equal efficacy; however, in the long term, HA was found to have an enhanced effect. No statistically significant difference was observed in the adverse events caused by the two interventions. Further investigation and understanding into the trend observed in the present study may aid clinicians in the treatment of knee OA.

## Introduction

Osteoarthritis (OA) of the knee is a common, chronic joint disorder, characterized by articular cartilage degeneration and secondary hyperosteogeny (1). OA often causes severe pain in the knee joint and affects 35% of individuals that are >65 years-old (2). There are an estimated 46 million (22%) adults in the USA suffering from OA (3). OA requires a variety of treatments to ease the pain and improve functioning. Currently, several therapeutic approaches are used, including rest, medication, other noninvasive interventions, nonsurgical invasive interventions and surgical interventions (4). However, if pain persists following rest or medication and other noninvasive interventions have failed, then prior to surgical intervention, intra-articular injections of a number of drugs may be administered. These typically include hyaluronic acid (HA), corticosteroids (CS) and diclofenac.

Intra-articular HA injections have been demonstrated to be beneficial in the treatment of OA, improving joint lubrication and synovial fluid viscosity, normalizing hyaluronan synthesis, inhibiting proteoglycan degradation and exhibiting analgesic and anti-inflammatory effects (5–8). However, the safety of HA remains controversial. Several studies (9–13) have revealed that the use of HA may cause an increased risk of serious adverse events and local adverse events, indicating that intra-articular HA injections should be discouraged.

Intra-articular injections of CS have been used for the past decade in the treatment of OA, and appear to be relatively safe. CS have anti-inflammatory effects due to the inhibition of inflammatory cytokines, as well as the inhibition of the pathways that lead to their functioning (14). However, the duration effect of CS is considerably less than the recommended interval between doses (15). Therefore, the short-term effects are acceptable; however, the long-term effects require further investigation.

Numerous systematic reviews have investigated the effects of HA and other placebos (9,16–18), or CS and placebos (19,20); however, there are few studies that have compared HA and CS (15). Thus, the aim of the present study was to conduct a meta-analysis to determine which treatment method was more effective, comparing intra-articular HA injection with CS, and to assess whether intra-articular HA injections were associated with a lower incidence of adverse events compared with CS.

## Materials and methods

### Selection of trials

By performing a comprehensive search based on electronic databases and manual literature searches, all the relevant randomized controlled trials (RCTs) comparing intra-articular injections of HA with CS until July 2013 were identified. The electronic sources included PubMed, EMBASE and The Cochrane Central Register of Controlled Trials. Search terms included ‘hyaluronic acid’, ‘hyaluronan’, ‘corticosteroids’, ‘glucocorticoids’, ‘knee osteoarthritis’ and ‘randomized controlled trials’. In addition, references of the relevant selected articles were reviewed to identify further articles. There were no language restrictions and no protocol for systematic review.

Articles were included if they met the following criteria: (i) Patients were ≥18 years-old and had symptomatic knee OA; (ii) random allocation of treatments; (iii) compared the therapeutic effects of intra-articular HA with intra-articular CS; (iv) follow-up time was ≥3 months; (v) inclusion of ≥1 valid outcome, including the visual analog scale (VAS), Lequesne index, range of motion of the knee, Knee Society Clinical Rating System (KSS) and adverse events; and (vi) outcomes were expressed as mean values or standard deviations (SD), and graphic outcomes as numerical values.

Articles reporting the result of intramuscular or oral drug interventions were excluded, as well as those whose outcomes compared HA/CS with other placebos.

### Data collection and analysis

Two independent authors searched the electronic databases based on the titles and abstracts of all the trials to identify relevant studies for inclusion. The authors independently completed the evaluation of the included trials. When the data were incomplete, the author was contacted for sufficient information. If the article did not meet the inclusion criteria, it was excluded from the analysis. If no agreement was reached, the senior author (Dr Xijing He) made the final decision. Data of selected articles were carefully cross-checked to ensure that no duplicate data had been presented.

### Data extraction

General characteristics, intervention or treatment methods and outcomes were recorded for each study included in the meta-analysis. General characteristics included the publication year, geographical location, participant number and demographics, follow-up time and study design. For all the included articles, the intervention methods were recorded, including the HA/CS dose regimen and frequency. The measured outcomes, as described in the inclusion criteria, were also observed, and all adverse events were recorded. If necessary, the authors were contacted for further information.

### Assessment of trial quality

Two authors independently assessed the methodological quality using the Jadad scoring system (21). The Jadad scoring system scored the trials methodologically according to the principle of randomization (0–2 points), blinding implementation (0–2 points) and the withdrawal and lost to follow-up (0–1 points) of the included studies. Using the Jadad scoring system, studies with a total score of 0–2 points were considered as low quality studies, while a total score of ≥3 points were considered high-quality research. The two authors tried to reach a consensus when there were divergences.

### Meta-analysis assessment of the treatment effects and outcomes

A meta-analysis of the data, including the effects and outcomes of the treatments, was performed using the Inverse Variance method with Review Manager software (RevMan version 5.0; Cochrane Collaboration, Freiburg, Germany). For continuous data, such as the VAS, mean ± SD values were used to calculate the weighted mean difference and 95% confidence interval (CI). For dichotomous data, relative risk (RR) and 95% CI were applied.

### Assessment of heterogeneity

Statistical heterogeneity was assessed using the χ^2^ test (22). A value of I^2^>50% was indicative of substantial heterogeneity, recommending a random effects modeling estimate. Otherwise, a fixed effects approach was used.

### Analysis of sensitivity

Reanalyzing the data using different statistical approaches, for example using a random effects model instead of a fixed effects model, was utilized for the sensitivity analysis of the meta-analysis.

## Results

### Included studies

**Figure 1:**
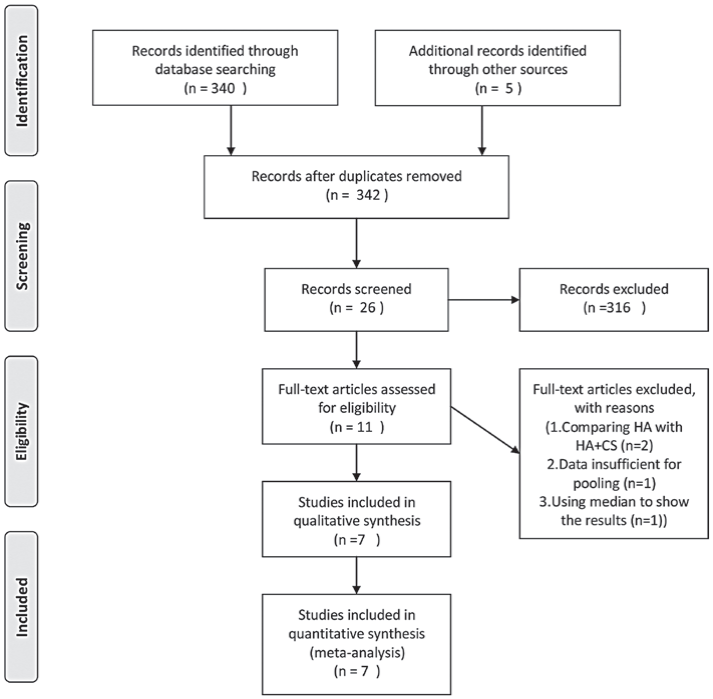
Summary of the search results and the selection procedure for inclusion.

An initial search yielded a total of 342 studies, of which 316 were excluded following title and abstract screening. The remaining 26 studies were fully retrieved, and 15 studies were excluded as they did not meet the inclusion criteria. A total of 11 RCTs fit the inclusion criteria (23–33). However, two of the trials assessed the efficacy of intra-articular HA with or without CS (31,32), one study reported only median values of the outcome results and had no mean values or measures of variance (26), while an additional study did not use a validated scale to report the outcomes (33). Therefore, the meta-analysis was performed on seven studies with a total 583 patients. A detailed flow chart for the selection is shown in Fig. 1.

### Quality of studies

For all the included studies, the methodological quality represented the risk of bias assessment. All the studies adopted the method of randomization (23–25,27–30) and the use of allocation concealment was unclear with the exception of one study (30). Three studies applied a double-blind technique (23,28,29), while two studies applied a single-blind study design (24,27). Only one study did not use a blinding method (25), indicating a potential selection bias. The quality scores of the included studies are shown in Table I.

### Study characteristics

A total of 583 participants (222 males and 361 females) were included from the seven studies. In total, 298 participants were administered an HA arm and 285 participants received CS. Of the seven studies used in the analysis, one compared Hyalgan with triamcinolone hexacetonide (23), one compared Hyalgan with methylprednisolone (24), one compared Orthovisc (Anika Therapeutics, Inc., Woburn, MA, USA) with methylprednisolone (25), one compared Synvisc with triamcinolone hexacetonide (27), one compared Durolane (Q-Med AB, Uppsala, Sweden) with triamcinolone (28), one compared Ostenil (TRB Chemedica AG, Haar/München, Germany) with triamcinolone (29) and the final study compared Na-HA (Artzdispo; Kaken Pharmaceutical, Tokyo, Japan) with Decadron (30). Details of the studies are shown in Table II.

### Meta-analysis

#### VAS of knee OA

**Figure 2:**
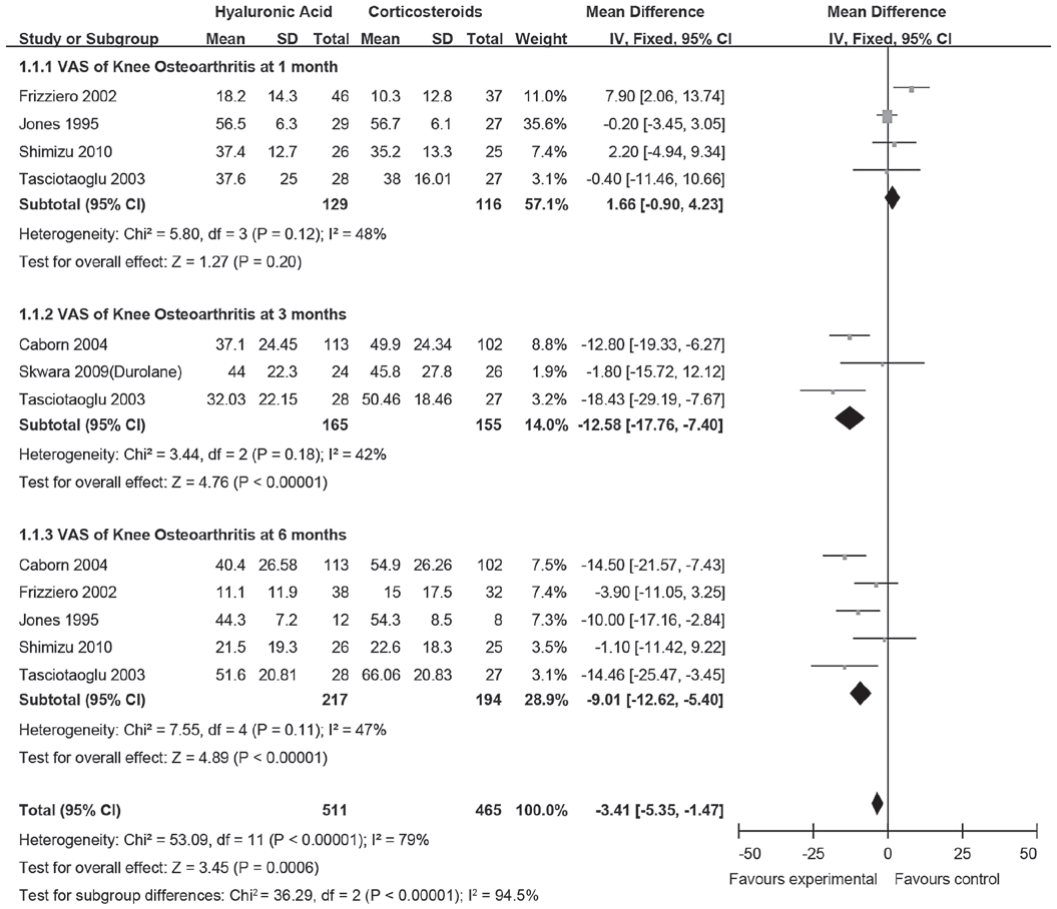
Forest plot diagram showing the VAS of knee OA. VAS, visual analog score; SD, standard deviation; CI, confidence interval; OA, osteoarthritis.

A total of four studies (23–25,30), including 129 HA and 116 CS-treated patients (n=245), reported detailed data on the VAS score of knee OA after one month of treatment. CS injections were found to reduce the VAS score by a mean of 1.66 mm compared with the HA group; however, the difference was not statistically significant (95% CI, −0.90, 4.23; P=0.20; I^2^=48%; Fig. 2).

A total of three studies (25,27,28), including 165 HA and 155 CS-treated patients (n=320), reported detailed data on the VAS score of knee OA after three months. HA injections significantly reduced the VAS score by a mean of 12.58 mm, ranging between 7.40 and 17.76, when compared with that in the CS group (95% CI; P<0.00001; I^2^=42%; Fig. 2).

A total of five studies (23–25,27,30), including 217 HA and 194 CS-treated patients (n=411), reported detailed data on the VAS score of knee OA after six months. HA injections were found to significantly reduce the VAS score by a mean of 9.01 mm, ranging between 5.40 and 12.62, when compared with that in the CS group (95% CI; P<0.00001; I^2^=47%; Fig. 2).

#### Lequesne index of knee OA

**Figure 3:**
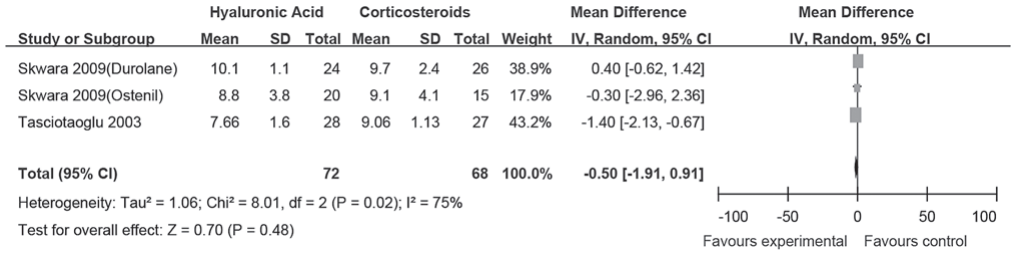
Forest plot diagram showing the Lequesne index of knee OA. SD, standard deviation; CI, confidence interval; OA, osteoarthritis.

Three studies (25,28,29), including 72 HA and 68 CS-treated patients (n=140), reported detailed data on the Lequesne index of knee OA after three months. No statistically significant differences in the Lequesne index were observed between the two groups (mean difference (MD), −0.50; 95% CI, −1.91, 0.91; P=0.48; I^2^=75%; Fig. 3).

#### KSS of knee OA

**Figure 4:**
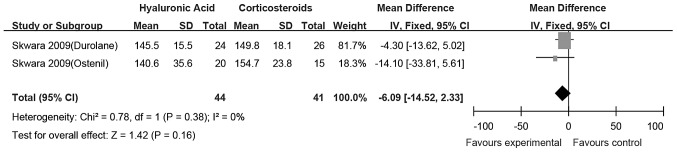
Forest plot diagram showing the KSS of knee OA. SD, standard deviation; CI, confidence interval; KSS, Knee Society Clinical Rating Score; OA, osteoarthritis.

Two studies (28,29), including 44 HA and 41 CS-treated patients (n=85), reported detailed data on the KSS of knee OA after three months. No statistically significant differences in the KSS were identified between the two groups (MD, −6.09; 95% CI, −14.52, 2.33; P=0.16; I^2^=0%; Fig. 4).

#### Maximum flexion of knee OA

**Figure 5:**
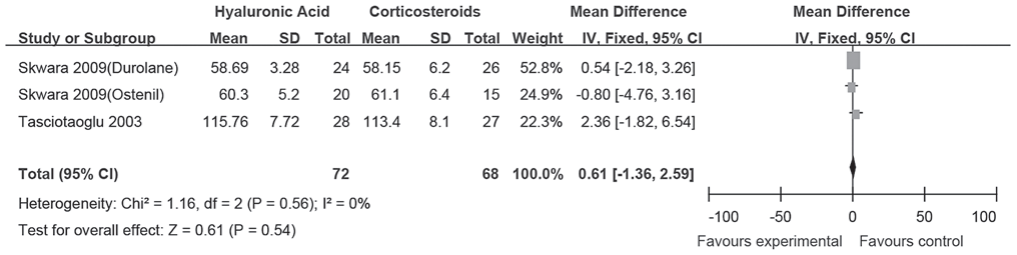
Forest plot diagram showing the maximum flexion of knee OA. SD, standard deviation; CI, confidence interval; OA, osteoarthritis.

Three studies (25,28,29), including 72 HA and 68 CS-treated patients (n=140), reported detailed data on the maximum flexion of knee OA after three months of treatment. No statistically significant differences in the maximum flexion were observed between the two groups (MD, 0.61; 95% CI, −1.36, 2.59; P=0.54; I^2^=0%; Fig. 5).

#### Adverse events of knee OA

**Figure 6:**
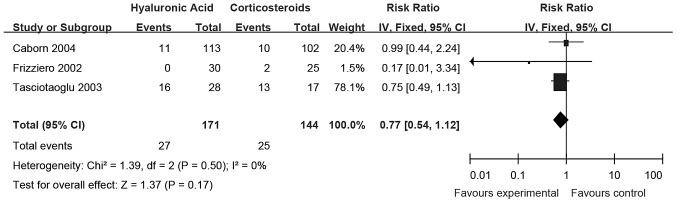
Forest plot diagram showing the adverse events of knee OA. SD, standard deviation; CI, confidence interval; OA, osteoarthritis.

In total, three studies (24,25,27), including 171 HA and 144 CS-treated patients (n=315), reported detailed data on the adverse events of knee OA. No statistically significant differences in the adverse events were observed between the two groups (RR, 0.77; 95% CI, 0.54, 1.12; P=0.17; I^2^=0%; Fig. 6).

### Publication bias analysis

**Figure 7:**
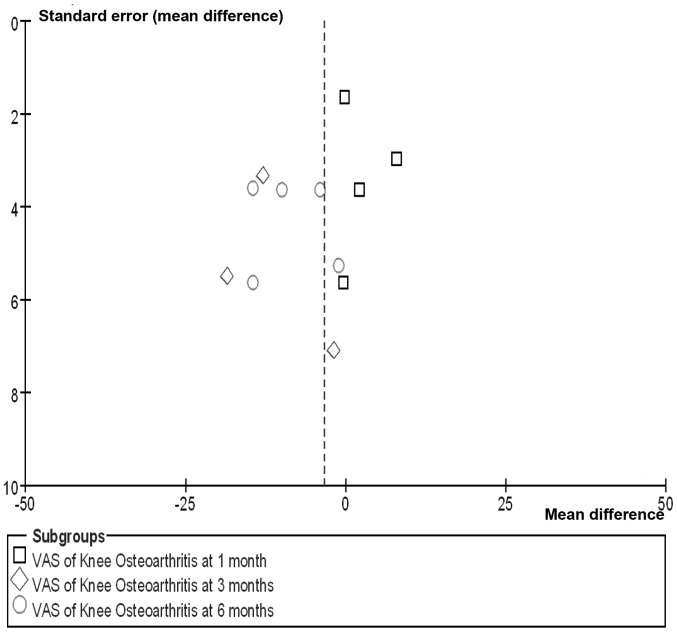
Funnel plot showing the publication bias of the subgroups with regard to VAS at the different time periods. VAS, visual analog score. SE, standard error; MD, mean difference.

The VAS of knee OA was used for the funnel plot analysis of publication bias (Fig. 7), which revealed there was no marked publication bias evident for the outcome.

## Discussion

In the present study, a meta-analysis of trials comparing HA with CS for the treatment of OA was performed, and the results indicated that the curative effect of the different treatments varied over time. With regard to the VAS of knee OA, the two drugs (HA and CS) appeared to be equally effective for pain in the short term (≤1 month). However, after ≥3 months, HA was found to have a greater relative effect compared with CS. By contrast, for other indicators, including the Lequesne index, KSS and maximum flexion, no statistically significant differences were observed between the two treatment approaches for knee OA. Similarly, for the adverse reactions, no difference was observed between the two drugs. However, this meta-analysis compared the two interventions on their efficacy in knee OA treatment and did not directly compare their efficacy with a placebo; thus, it is important to determine whether HA and CS are effective in the treatment of OA according to recent studies (2,16,20,35). The results indicate that the therapeutic effect of HA may be longer lasting compared with CS.

Bannuru *et al* (15) also performed a meta-analysis of systematic reviews analyzing the efficacy of intra-articular HA in the treatment of OA compared with intra-articular CS. However, only VAS was used as an evaluation index and all the trials included were published prior to 2004. Despite these differences, the conclusions of the meta-analysis were in accordance with the results of the present study.

One unique feature of the present meta-analysis was that the therapeutic response was based on time by separately pooling the data for each time point. The pattern of the therapeutic response was then attributed to different interventions to a large extent. However, not all the trials had the full clinical data associated with each of the time points. Therefore, the data made available were collected for comparative analysis. Other similar prospective RCTs exist; however, the complete data were not available.

In order to minimize publication bias, a large search strategy was used independently by two reviewers. The funnel plot (Fig. 7) indicated that there was no marked publication bias with regard to the selection of trials included in the meta-analysis. The reliability on the risk of bias assessment and data extraction was enhanced as the two authors performed the procedures independently, from which a consensus was then obtained. In addition, the bias was minimized through study design and quality by performing sensitivity analyses based on the study design, which provided an overview of the risk of bias assessment.

An additional problem may have been the use of Jadad scores to evaluate the quality of the trials. The majority of the studies included in the meta-analysis had Jadad scores greater than three, and a number had full Jadad scores, indicating that the majority of the articles were of high quality. However, the Jadad scoring system has been described as simplistic since it takes into account a limited number of variables and does not consider bias as a result of allocation concealment (36). Therefore, the Pedro format (www.cchs.usyd.edu.au/pedro/), which includes more variables, was used to further assess the quality of the trials.

The safety of the two interventions was also investigated. The majority of the injections were performed at an injection site above the tibial plateau and lateral to the patellar tendon, with the knee flexed at ~90° and without ultrasound or fluoroscopy guidance. Notably, the adverse effects were rare or insignificant (37,38), and the occurrence rate was not significantly different between the two interventions. The most common adverse effects were arthralgia, injection site pain, joint swelling and injection site edema (27). No joint space loss was observed at the knee joint following interventions in OA; thus, we hypothesize that clinical operators should be careful when the injections are performed in order to alleviate the discomfort of the procedure, localized pain post-injection and flushing.

There were certain limitations in the present. Firstly, the number of included trials was limited, which may have resulted in insufficient significant effectiveness. In addition, the meta-analysis suffered from the pooling of a variety of HA agents that differed with regard to characteristics, including molecular weight, origin, viscosity and cross-linking. To avoid this type of bias, sensitivity analyses may be performed. However, sensitivity analysis based on the viscosity or molecular weights was not performed since this may have biased the review as a direct comparison between different agents. Furthermore, sensitivity analyses based on comparing the same type of HA agent with one type of CS agent were not successful, primarily due to the paucity of the data.

In conclusion, the results of the meta-analysis demonstrated that HA has a similar level of pain relief compared with CS in the short term (up to one month); however, HA is more effective than CS over a longer time period (up to six months). The potential for adverse events are similar between the two interventions. Understanding the length of the clinical efficacy and adverse events of these two drugs is useful for clinicians to produce a therapeutic regimen for OA patients. However, more high-quality RCTs with long-term follow-ups and large sample sizes are required in the future.

## Figures and Tables

**Table I:** Jadad quality scores of the seven studies of knee OA included in the meta-analysis.

Study	1	2	3	4	5	6	7	8	9	10	11	Jadad score
Jones 1995 (19)	+	+	?	+	+	−	+	−	−	+	+	5/5
Frizziero 2002 (20)	+	+	?	+	−	−	+	+	+	+	+	3/5
Tasciotaoglu 2003 (21)	+	+	?	+	−	−	−	+	−	+	+	2/5
Caborn 2004 (23)	+	+	?	+	−	−	+	+	+	+	+	5/5
Skwara 2009 (Durolane) (24)	+	+	?	+	+	+	−	−	−	+	+	5/5
Skwara 2009 (Ostenil) (25)	+	+	?	+	+	+	−	−	−	+	+	5/5
Shimizu 2010 (26)	+	+	+	+	?	?	?	−	−	+	+	3/5

Numbers 1–11 follow the Pedro format, and the Jadad score was calculated from the different set of criteria. 1, eligibility criteria specified; 2, patients were randomized to the groups; 3, concealment of allocation; 4, groups were similar at the baseline; 5, patients were blinded; 6, practitioners administering the intervention were blinded; 7, assessors were blinded; 8, measurements of the key outcomes obtained from >85% of patients; 9, intention to treat analysis; 10, statistical comparisons between groups; 11, point measures and measures of variability provided; +, criteria clearly satisfied; −, criteria not clearly satisfied; ?, unclear whether criteria was satisfied.

**Table II:** Characteristics of studies included in the meta-analysis.

Study, location	Patients (n)	Gender (M:F)	Mean age (years)	Follow-up time	Characteristics of the participants	Interventions	Outcomes analyzed
Jones 1995 (19), UK	63	24:39	70.5	6 months	Bilateral knee OA with bilateral effusions	Exp: 32, 20 mg Hyalgan 5-weekly injections; Ctl: 31, 20 mg triamcinolone hexacetonide single injections followed by 4 placebo injections	VAS; duration of stiff-ness; ROM; joint effusion; local heat; synovial thickening; joint-line and peri-articular tenderness
Frizziero 2002 (20), Italy	99	46:53	49.5	6 months	Kellgren-Lawrence grades I–III; fulfilling the clinical and radio-logical criteria of the American College of Rheumatology (31)	Exp: 52, 2 ml (20 mg) Hyalgan 5-weekly injections; Ctl: 47, 1 ml (40 mg) methylpred-nisolone 3-weekly injections	Arthroscopic findings; VAS; morning stiff-ness; maximum active extension and flexion
Tasciotaoglu 2003 (21), Turkey	60	0:60	59	6 months	Kellgren-Lawrence grade II–III knee OA radiologically; VAS of >40 mm	Exp: 30, 2 ml (30 mg) Orthovisc 3-weekly injections; Ctl: 30, 1 ml (40 mg) methylpred-nisolone 3-weekly injections	VAS; Lequesne index; functional index; range of knee lexion; adverse events
Caborn 2004 (23), USA	218	95:123	63.1	26 weeks	Diagnosed with OA ≥3 months	Exp: 113, 2 ml (16 mg) Synvisc 3-weekly injections; Ctl: 105, 2 ml (40 mg) triamcinolone hexacetonide single injection	WOMAC scores; VAS
Skwara 2009 (Durolane) (24), Germany	50	27:23	61	12 weeks	Kellgren-Lawrence grade II–III knee OA radiologically; VAS of >40 mm; persistent pain for ≥6 months; Lequesne score ≥10; good compliance	Exp: 24, 3 ml (60 mg) Durolane single injection; Ctl: 26, 1 ml (10 mg) triamcinolone single injection	Gait pattern (ROM of knee and hip); muscle activity; VAS; Lequesne index; KSS; SF-36
Skwara 2009 (Ostenil) (25), Germany	42	17:25	61	12 weeks	Kellgren-Lawrence grade II–III knee OA radiologically; VAS of >40 mm; persistent pain for ≥6 months; Lequesne score ≥10; good compliance	Exp: 21, 2 ml (20 mg) Ostenil 5-weekly injections; Ctl: 21, 1 ml (10 mg) triamcinolone 5-weekly injections	Gait pattern; muscle activity; VAS; Lequesne index; KSS; SF-36
Shimizu 2010 (26), Japan	51	13:38	>60	6 months	OA findings on radiography and Kellgren-Lawrence grade II or III; persistent pain for ≥6 months; hydro-; arthrosis; no treatment within 3 months	Exp: 26, 25 mg Na-HA 5-weekly injections; Ctl: 25, 4 mg Decadron single injection	Pain/inflammation scores; VAS; joint fluid levels (HA, MMP-9, TIMP-1); Gotoh score

Exp, experimental group; Ctl, control group; VAS, visual analog score; ROM, range of motion; HA, hyaluronic acid; MMP, matrix metalloproteinases; TIMP, tissue inhibitors of metalloproteinases; WOMAC, Western Ontario and McMaster osteoarthritis index; OA, osteoarthritis; M, male; F, female; SF-36, 36-Item Short Form Health Survey; KSS, Knee Society Clinical Rating System. The number after Exp and Ctl refers to the number of patients in each group.
